# Prevalence of anemia and associated factors among pregnant women in Southern Ethiopia: A community based cross-sectional study

**DOI:** 10.1371/journal.pone.0188783

**Published:** 2017-12-11

**Authors:** Meaza Lebso, Anchamo Anato, Eskindir Loha

**Affiliations:** 1 Yirgalem Hospital, Department of Medical Laboratory, Yirgalem, Ethiopia; 2 School of Nutrition, Food Science and Technology, College of Agriculture, Hawassa University, Hawassa, Ethiopia; 3 School of Public and Environmental Health, College of Medicine and Health Sciences, Hawassa University, Hawassa, Ethiopia; Oklahoma State University, UNITED STATES

## Abstract

**Background:**

Anemia is defined as a condition in which there is less than the normal hemoglobin (Hb) level in the body. During pregnancy; iron deficiency is associated with multiple adverse outcomes for both mother and infant. Most of the studies conducted in Ethiopia on anemia during pregnancy were conducted at institution level and associated factors are not well studied and documented. Independent factors like, food security status, dietary diversity and intestinal parasites infection were considered by only a few of them. Hence, the aim of this study was to determine the prevalence of anemia and associated factors among pregnant women in Lemo District, Southern Ethiopia.

**Methods:**

Community based cross- sectional study was conducted from May-June 2015. Multistage sampling was used to include 507 study participants. Anaemia was diagnosed using HemoCue HB 301 and haemoglobin concentration <11 g/dl was classified as anaemic. Stool examinations were also done. Structured questionnaire was used as a tool to collect sociodemographic characteristics, individual dietary diversity and level of household food security data. Multivariate logistic regression model was employed to determine the effect of explanatory variables like level of education, level of household food security, dietary diversity, trimester of pregnancy, family planning before pregnancy, deworming, gravidity, iron intake in current pregnancy and soil transmitted helminthes on dependent variable anemia.

**Results:**

The prevalence of anemia was 23.2% (95% CI: 19.5%-26.9%). Factors associated with anemia were: low socio-economic status (AOR = 2.03, 95% CI: 1.11–3.69), trimester second (AOR = 3.09, 95%CI: 1.41–6.79) and third (AOR = 3.68, 95% CI: 1.67–8.08), gravidity three to five (AOR = 1.78, 95% CI: 1.03–3.07) and six and above (AOR = 2.59, 95%CI: 1.37–4.92), not supplemented with iron (AOR = 1.72, 95% CI: 1.02–2.91), low dietary diversity score (AOR = 3.18, 95% CI: 1.37–7.37) and hookworm infection (AOR = 2.69, 95%CI: 1.34–5.39).

**Conclusion:**

Anemia has moderate public health significance in the area. Community-based interventions should be enhanced considering the identified associated factors.

## Introduction

Anemia is a condition characterized as a low level of hemoglobin in the blood, as evidenced by a reduced quality or quantity of red blood cells which decreases oxygen-carrying capacity to tissues [1,2]. It occurs at all age groups, but is more prevalent in pregnant women and children [3]. Even in normal pregnant women, the hemoglobin concentration decreases with dilution as the volume of circulating blood increases [4].

Globally 1.62 billion people (25%) are anemic, among which 56 million are pregnant women [5]. Around 40% of women begin their pregnancy with low or absent iron stores (serum ferritin <30 mg/l) and up to 90% have iron stores of <500 mg (serum ferritin <70 mg/l) worldwide, which is insufficient to meet the increased iron needs during pregnancy and postpartum [6]. In Africa the prevalence of anemia among pregnant women was 57.1% [2] which is associated with adverse health outcomes for both mother and infant, like maternal mortality, perinatal mortality, growth restriction and low birth weight [7, 8,9].

As one of the developing countries, Ethiopia shares the burden of the problems of anemia. According to EDHS’s report in 2012, 17% of Ethiopian women aged 15–49 were anemic, with 13% having mild anemia, 3% having moderate anemia, and 1% having severe anemia [10]. Despite the magnitude of the problem, there is scarcity of data on prevalence and associated factors among pregnant women in Southern Ethiopia. Therefore this study was conducted to assess the prevalence of anemia and its associated factors among pregnant women in Lemo district Southern Ethiopia.

## Materials and methods

### Study design, population and sampling procedures

Community based cross-sectional study was conducted in Lemo district, Southern Ethiopia from May-June 2015. A total of 507 pregnant women were included in the study. Multi-stage sampling technique was employed to select study participants. Lemo district is located in Southern Ethiopia with altitude from 1501–2500 meters above sea level. There are seven health centres and thirty five health posts in the district. The total population were 156, 267 and of them, 79,122 were women. There are 35 *Kebeles* in the district (*Kebele* is the lowest administrative unit in Ethiopia). Out of this, 10 (30%) *Kebeles* were selected by random sampling method. The sample size was distributed to each of the selected *Kebele* proportional to their population size. The sampling frame was prepared for each *Kebele* after identifying pregnant women through rapid registration by using house- to- house visits. The sampling interval was determined by dividing the total population in each *Kebele* to the size of the sample required from that *Kebele*. Pregnant women were then selected using systematic random sampling technique from the sampling frame prepared. The selected women were mobilized to the nearest health post with the help of health extension workers for data collection.

### Data collection tools and procedures

Data were collected using pretested structured questionnaire by face-to-face interview. Sociodemographic, economic, maternal dietary practices questionnaires were adopted from CSA and ORC Macro, 2011 [10] and modified in to that specific area’s context. Data collectors were health professionals with diploma and there were two supervisors. Two days intensive training was given for both data collectors and supervisors prior to the actual data collection.

### Individual blood sample collection and analysis

Hemoglobin analysis was carried out in the health post located in each *Kebele* by laboratory technologists. The haemoglobin concentration was measured by taking a finger-prick blood sample of each pregnant woman using a HemoCue Hb 301 (HemoCue AB, Angelholm, Sweden). A prick was made on the tip of the middle finger after the site was cleaned with disinfectant. The first drop of blood was cleaned off and the second drop was collected to fill the microcuvette which is then placed in the cuvette holder of the device for measuring hemoglobin concentration. The performance of the meter was checked on the daily bases by using control standard to increase test reliability. First the microcuvette holder was pulled to its loading position and when the meter was ready to use capillary blood, sample for examination filled in one continuous process. Within 10 minutes of filling, it was placed in the holder and pushed into its measuring position. Finally after 15–60 seconds, the result was displayed and recorded using WHO’s field surveys recommendation techniques [11].

Hemoglobin level was divided into three categories: 10–10.9 g/dl mild anemia, 7–9.9 g/dl moderate anemia and < 7g/dl severe anemia. Hemoglobin level was adjusted for altitude by using the following formula [12] before the data were entered. Hb adjustment = -0.032 x (altitude x 0.0032808) + 0.022 x (altitude x 0.0032808)^2^.

### Stool specimen collection and examination

The stool examination was conducted by using un-portable microscope in the health posts in each *Kebele*. After orientation was given to the women on how to collect sufficient amount and contamination free stool specimen, labeled clean stool specimen container was given to each pregnant woman with clean applicator stick. Two stool smears were prepared for each pregnant woman using saline solution for direct microscopic identification of intestinal parasites. The direct smear was examined by 10x and 40x microscopic magnifications with standard procedures within two hours of sample collection.

### Dietary, food security and wealth assessment

Women dietary diversity scores were computed based on FAO guidelines [13]. The score was calculated and converted into tertiles (low, medium and high). All of the reported foods and beverages consumed in a day before the survey by the pregnant women were categorized into nine food groups: cereals/starchy staples, oils/fat, dark green leafy vegetables and vitamin A rich fruits and vegetables, legumes, nuts and seeds, other fruits and vegetables, meat and fish, organ meat, milk and products and egg. A Participant who had consumed (at least once) the food within each sub group was scored 1 unless 0 was given.

The household food security questionnaires were adopted from FANTA version 3 and modified for local context; it contains 18 questions [14]. The first nine questions were answered as yes or no, and the scores were classified into one of the four categories: food secured, mildly food insecure, moderately food insecure and severely food insecure.

The wealth index was constructed using 19 variables related to ownership of selected household assets using a principal components analysis adopted from [10]. Some of the them are source of drinking water, kind of toilet facility, household facility (radio, television, mobile phone, table etc.), type of fuel, materials of floor, materials of exterior walls, materials of roof, number of rooms, ownership of agricultural land and its size, ownership of livestock and number, bank or microfinance saving etc. During analysis the four variables (radio, television, mobile phone and agricultural land) were dropped as their composite scores were less than 50%. Ultimately, the ten components with eigenvalues greater than one were identified and retained. The components explained 67.2% of the total variance which was above the recommended minimum value of 60% [15]. The wealth index values were calculated by summing up the scores of ten components. Finally, the three socio-economic categories (low, medium and high) were generated by splitting the wealth index values into three equal classes

### Data processing and analysis

Data was coded and entered into SPSS version 20 for analysis. Categorical type of data was analysed by descriptive statistics (frequency and percentage) whereas range, mean and standard deviation were used to present continuous variables.

With the intention of building one multivariate model, the criterion p<0.25 was used to select candidate variables. Meanwhile, based on Hosmer and Lemeshow rule of ten (10 cases per independent variable) [16], the number of variables to be used in the multivariate binary logistic regression model was eleven variables were used to develop multivariate binary logistic regression model. Out of the candidate variables, seven had p<0.05 and the rest four had p<0.25 in the binary logistic regression model. Prior to running the multivariate binary logistic regression, correlation among the predictor variables was checked to determine if the predictors were correlated. Accordingly two variables, total number of live birth and gravidity showed multicollinearity. Due to this, total number of live birth was not incorporated in the fitted model. Hosmer and Lemeshow goodness-of-fit statistics verified (p = 0.73) the fitted model. Finally odds ratio with 95% confidence interval was reported.

### Ethical considerations

Ethical clearance was obtained from Hawassa University Ethical Review Board and the ethics committee waived the requirement for parent/guardian consent for minors (aged 15–17 year old). Permission letter was obtained from Lemo district health office. Blood collection was done after obtaining a written consent from each study participant. Pregnant women who had anemia (Hb<11g/dl) were provided with iron-folate tablets and those who were in the third trimester and infected with intestinal parasites were given Albendazole tablet.

## Results

### Sociodemographic and economic characteristics

Of 507 study participants, 504 pregnant women had participated in the study, with a response rate (99%). The mean age ± (standard deviation) of the participants was 28.7 ± (5.8) years. The mean family size was 4.8 ± (1.8). Half of the participants 251(49.8%) were in the age range of 25–34 years, 444(88.1%) were housewives and 390 (77.4%) used to walk barefoot sometimes (Table 1).

**Table 1:** Socio-demographic and economic characteristics of pregnant women in Lemo district, Southern Ethiopia, 2015 (n = 504).

Variables (n = 504)	n	%
Age group		
15–24	154	30.6
25–34	251	49.8
35–49	99	19.6
Level of education		
Primary	319	63.3
Secondary	41	8.1
More than secondary	20	4.0
Non-educated	124	24.6
Occupation		
Housewife	444	88.1
Farmer	13	2.6
Merchant	36	7.1
Government employee	11	2.2
Wealth index		
Low	168	33.3
Middle	168	33.3
High	168	33.3
Walking with barefoot		
Sometimes	390	77.4
Always	67	13.3
Not at all	47	9.3

### Dietary practice and intestinal parasitic infection

**Fig 1:**
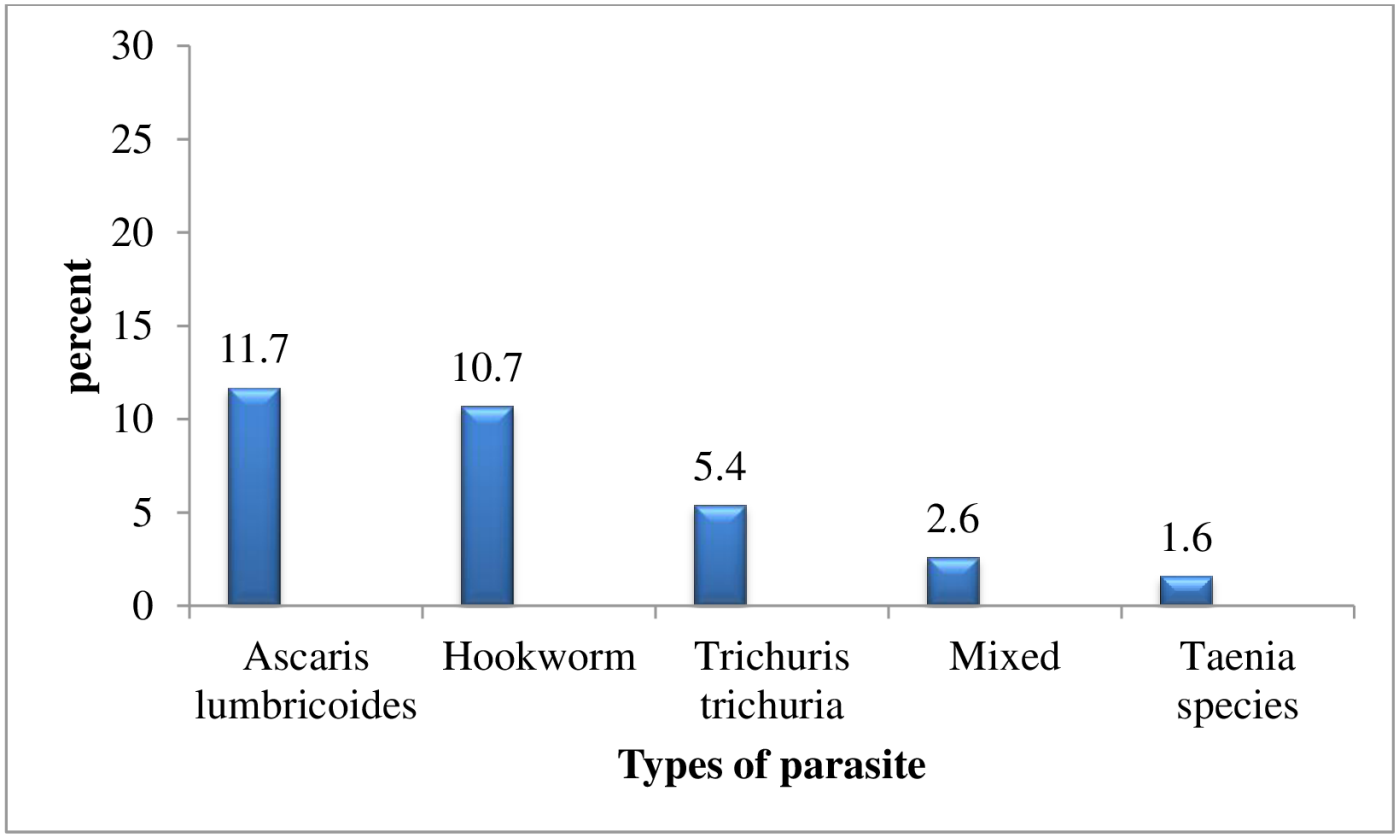
Prevalence of intestinal parasites among pregnant women in Lemo district, Southern Ethiopia, 2015 (n = 504). Mixed = *co—infection with one or more intestinal parasites*, *other = Taenia species and Trichuris trichuria*.

Majority, 468 (92.9%) of the pregnant women consumed cereals followed by dark green leafy vegetables and vitamin A rich fruits and vegetables 261 (51.8%) in the previous 24 hour priory to survey. Around half of the pregnant women, 237 (47%) had low dietary diversity score and 67 (13.3%) had a high dietary diversity score (Table 2). Moreover, 161 (31.9%) of the pregnant women were infected by intestinal pararites. The leading parasitic infection among pregnant women in the area was *Ascaris lumbricoide* 59 (11.7%) followed by Hookworm 54 (10.7%) (Fig 1).

**Table 2:** Dietary practice and level of food security among pregnant women in Lemo district, Southern Ethiopia, 2015 (n = 504).

Food groups	n	%
Cereal / starchy staples	486	96.4
Other fruits and vegetables	180	35.7
Dark green leafy vegetables and vitamin A rich fruits and vegetables	261	58.8
Organ meat	29	5.8
Egg	56	11.1
Meat and fish	49	9.7
Legumes, nuts and seeds	238	47.2
Milk and milk product	149	29.6
Oils and fats	236	46.8
DDS*		
High	67	13.3
Medium	200	39.7
Low	237	47.0
Level of food insecurity		
Food secured	108	21.4
Mildly food insecure	90	17.9
Moderately food insecure	210	41.7
Severely food insecure	96	19.0

**Key**: DDS* = Dietary diversity score

### Prevalence and severity of anemia

The overall prevalence of anemia was 117 (23.2%) with 95%CI (19.5–26.9). Of the anemic pregnant women, 78 (66.6%) had mild and 39 (33.3%) moderate anemia but there was no severe anemia. The mean (±) SD hemoglobin concentration among the study participants was 11.9± (1.4). The prevalence of anemia with respect to the trimester were first 11 (11.5%), second 50 (23.8%) and third 56 (28.3%).

### Factors associated with anemia

Adjusting for household monthly income, level of food security, availability of latrine, frequency of meal per day, eating animal source of food at least once per week, history of malaria infection, and nutritional status; low socio economic class (AOR 2.03, 95% CI: 1.11–3.69), trimester second (AOR 3.09, 95% CI: 1.41–6.79) and third (AOR 3.68, 95% CI: 1.67–8.08), gravidity three to five (AOR 1.78, 95% CI: 1.03–3.07) and six and above (AOR 2.59, 95% CI: 1.37–4.92), iron not supplemented (AOR 1.72, 95% CI: 1.02–2.91), hookworm infection (AOR 2.69, 95% CI: 1.34–5.39) and low dietary diversity score (AOR 3.18, 95% CI: 1.37–7.37) showed statistically significant association (p<0.05) with anemia (Table 3).

**Table 3:** Factors associated with anemia among pregnant women in Lemo district, Southern Ethiopia, 2015 (n = 504).

Variables	Anemia		COR(95%CI)	AOR(95%CI)
	Yes	No		
	n(%)	n(%)		
Wealth index				
Rich	26	142	1	1
Medium	42	126	1.82(1.06–3.14)*	1.66(0.90–3.05)
Poor	49	119	2.25(1.32–3.84)	2.03(1.11–3.69)*
Level of education				
Educated	85	302	1	1
Illiterate	39	78	1.78(1.13–2.72)*	1.35(0.80–2.26)
Level of food insecurity				
Food secured	22	86	1	1
Mildly food insecure	16	74	0.85(0.41–1.73)	0.79(0.36–1.73)
Moderately food insecure	58	152	1.49(0.85–2.61)	1.34(0.71–2.51)
Severely food insecure	21	75	1.09(0.56–2.15)	0.96(0.45–2.07)
DDS				
High	8	59	1	1
Medium	30	170	1.30(0.57–2.99)	1.20(0.49–2.93)
Low	79	158	3.69(1.68–8.09)*	3.18(1.37–7.37)*
Animal source food				
Yes	42	198	1	1
No	75	189	1.87(1.22–2.87)*	1.48(0.91–2.41)
Trimester of pregnancy				
First	11	85	1	1
Second	50	160	2.42(1.19–4.88)*	3.09(1.41–6.79)*
Third	56	142	3.05(1.51–6.14)*	3.68(1.67–8.08)*
Gravidity				
<3	30	164	1	1
3–5	54	155	1.91(1.16–3.13)*	1.78(1.03–3.07)*
≥ 6	33	68	2.65(1.50–4.69)*	2.59(1.37–4.92)*
Family planning before pregnancy				
Yes	42	170	1	1
No	75	217	1.39(0.91–2.15)	1.44(0.89–2.33)
Iron intake in current pregnancy				
Yes	31	132	1	1
No	86	255	1.44(0.91–2.28)	1.72(1.02–2.91)*
Deworming				
Yes	18	85	1	1
No	99	302	1.55(0.89–2.70)*	1.81(0.97–3.38)
STH				
No ova / parasite	64	279	1	1
Hookworm	21	33	2.77(1.51–5.11)*	2.69(1.34–5.39)*
*Ascaris lumbricoides*	18	41	1.91(1.03–3.55)*	1.62(0.82–3.21)
Others	14	34	1.79(0.91–3.54)	1.84(0.87–3.89)

1 = reference, STH = soil transmitted helminths, DDS = dietary diversity score.

*statistically significant association (p<0.05).

AOR = adjusted odds ratio, COR = crude odds ratio

## Discussion

The study finding showed that the overall prevalence of anemia was 23.2%. Out of which 78 (66.6%) had mild anemia and 39 (33.3%) had moderate anemia but there was no severe anemia. According to WHO classification the prevalence of anemia among pregnant women in this study was a moderate public health problem [3]. The prevalence determined in this study was comparable with the other studies conducted in Ethiopia [17] and the national prevalence [10] that reported 21% and 22%, respectively. But it was lower as compared to other studies carried out in different parts of Ethiopia that were reported (32.8%) [18], (36.6%) [19] and (39.9%) [20]. These differences in the prevalence of anemia might be due to socio-economic variations, cultural and dietary patterns across regions within the same country.

Socio-economic status of household was significantly associated with anemia among pregnant women. Women from lower socio-economic class had higher prevalence of anemia than those from higher socio economic status. Our finding was consistent with other studies conducted in Nigeria and India, where higher prevalence of anemia was reported in lower socio-economic class [21,22]. This may be due to those women from lower socio-economic status being unable to purchase the good quality as well as enough quantity of foods.

The other variable that showed significant association with anemia in this study was gestational age. Women in the second and third trimester were 3.09 and 3.68 times more likely to be anemic than those who were in the first trimester. Similar studies conducted in different parts of Ethiopia have reported that women in the second and third trimester were at greater risk of developing anemia compared to those in the first trimester [23,24,25]. Similarly other studies done in India found that the prevalence of anemia was higher in pregnant women in the second and third trimesters [26]. This might be due to the fact that during pregnancy the need for calorie and nutrients are increased to support increased maternal metabolism, blood volume and the delivery of nutrients to the fetus [27] and this demand more increases in the second and third trimesters of pregnancy. In the first trimester there is a marked decrease in the absorption of iron probably because of lower iron requirements and menstruation stops, saving median of 0.56 mg Fe/day (160 mg/pregnancy) [28]. However, in the second trimester iron absorption from a diet of very high iron bioavailability increases by 1.9mg/day and in the last trimester it increases by up to 5.0 mg/day [29].

Intestinal parasites particularly hookworm infection has long been recognized among the major causes of anaemia in poor communities. Evidence shows that low coverage of anthelmintic treatment in maternal health programmes in many countries has been the result and increased hookworm infection intensity is associated with lower haemoglobin levels in pregnant women. It is also estimated that between a quarter and a third of pregnant women in sub-Saharan Africa are infected with hookworm and at risk of preventable hookworm-related anaemia [30].

In the current study women who were infected with hookworm were 2.69 times more likely to be anemic than who were not infected by any intestinal parasite. Our finding was in agreement with different studies conducted in different parts of Ethiopia and other country that reported higher odds of anemia among hookworm-infected women [31, 17,32]. Additionally, similar studies also showed higher odds of anemia among those having co-infection of hookworm with other intestinal parasites [33, 34,35]. The worm in the intestine may cause intestinal necrosis and blood loss as a result of the attachment to the intestinal mucosa and chronic infections lead to iron deficiency and anemia resulting from the excessive loss of iron [3].

Therefore, an effective intervention packages need to reduce anaemia among pregnant women through iron supplementation, anthelmintic treatment and dietary diversification in the study area. The study finding reported that combined supplement of deworming and iron has a high impact on haemoglobin than deworming alone supports the assertion that deworming is unlikely to replenish iron stores in the short period of time, and needs to be combined with iron supplementation, especially among communities whose diets is low in bioavailability of iron [36].

Moreover, this study showed that women who had low dietary diversity score were 3.18 times more likely to develop anemia than those with higher dietary diversity score. This finding was consistent with previous studies conducted in Ethiopia [37,38] which were showed that women who had low dietary diversity score and not consuming iron rich food were more likely to develop anemia than their counter parts. Similarly, in Ethiopia, women with restricted dietary behavior were more likely to be anemic compared to those without restrictive dietary behavior [23]. Studies conducted in Pakistan and Turkey also reported that consumption of fruits two or more time per week was associated with decreased risk of anemia [39,40]. Moreover, other study from Ghana showed that high maternal dietary diversity was associated with reduced risk of anaemia and so nutritional factors may be important [41]. This might be potential evidence that increase in individual dietary diversity score is related with increased nutrient adequacy and it can be used as a proxy indicator for measuring nutrient adequacy among pregnant females [42].

Cross-sectional surveys are the main means of measuring the burden of health problems but they have limitation in revealing the temporal sequence between the factors and the outcome variable. Moreover, micronutrients like folate, vitamin B12 and vitamin A were not assessed in this study.

In conclusion, iron deficiency anemia has a moderate public health concern among pregnant women in Lemo District, Southern Ethiopia. The factors associated with anemia in this study population were Socio-economic status, gravidity, trimester, iron supplementation, dietary diversity score and hookworm infection. The observed prevalence of anemia might affect the birth outcome of pregnant women. Enhancement of dietary practices, iron supplementation, promotion of family planning and treatment of intestinal parasites are specially increased efforts should be made to increase the coverage of anthelmintic treatment among pregnant women in this community.
